# Thymoma with acute gastric volvulus: a case report

**DOI:** 10.1186/s12885-017-3802-7

**Published:** 2017-11-29

**Authors:** Ailing Liu, Xin Gao, Lin Zhao

**Affiliations:** 1 Department of Oncology, Peking Union Medical College Hospital, Chinese Academy of Medical Sciences and Peking Union Medical College, Beijing, 100730 China; 2 Department of Radiology, Peking Union Medical College Hospital, Chinese Academy of Medical Sciences and Peking Union Medical College, Beijing, 100730 China

**Keywords:** Acute gastric volvulus, Thymoma, Phrenic nerve palsy

## Abstract

**Background:**

Acute gastric volvulus (GV) is a rare disease with high mortality rate often associated with anatomic abnormalities. Thymoma is the most common neoplasm located in the anterior mediastinum. There is no reported relationship between thymoma and GV. Here we reported a case of thymoma with initial symptom of acute gastric volvulus.

**Case presentation:**

A 43-year-old man complained of postprandial abdominal pain, nausea and vomiting. Acute gastric volvulus was diagnosed by chest radiograph, upper digestive tract radiograph and CT scan; later type B3 thymoma was diagnosed by biopsy of mediastinal mass. We inferred that gastric volvulus was secondary to thymoma due to phrenic nerve palsy. The patient was treated with endoscopic de-rotation. Further radiotherapy and chemotherapy were given. During treatments, GV still occurred with less severity and a reduced frequency of approximately every three to four months.

**Conclusion:**

We report the first case of thymoma initially presented with acute GV. We suspect a pathological mechanism related to the phrenic nerve palsy. This case indicates that thymoma may present alongside rare acute GV.

## Background

Gastric volvulus (GV) is a rare condition resulting from rotation of the stomach more than 180 degrees [1]. It is recognized to be life-threatening thus prompt diagnosis and treatment is imperative.

GV is most often found in the elderly, with a peak incidence around 50 [2]. Based on etiology, GV can be divided into primary and secondary. The first, occurring in 30% of cases, is associated with congenital defects leading to absence or laxity of the supporting structures of the stomach (such as gastrocolic, gastro-hepatic ligaments). Secondary GV is more common and usually associated with other anatomic abnormalities, such as paraesophageal hernia, diaphragmatic hernia, phrenic nerve paralysis, et al. [3].

Though thymoma is the most common tumor of the anterior mediastinum, it is extremely rare [4]. Approximately one third of patients with thymoma are asymptomatic, most of them in early stage. In symptomatic patients, main clinical manifestations include cough, chest pain, phrenic nerve palsy and superior vena cava syndrome [5]. No incident of a thymoma patient presenting with gastric volvulus has been reported.

Here we report a case of thymoma with initial symptom of acute gastric volvulus.

## Case presentation

**Fig. 1:**
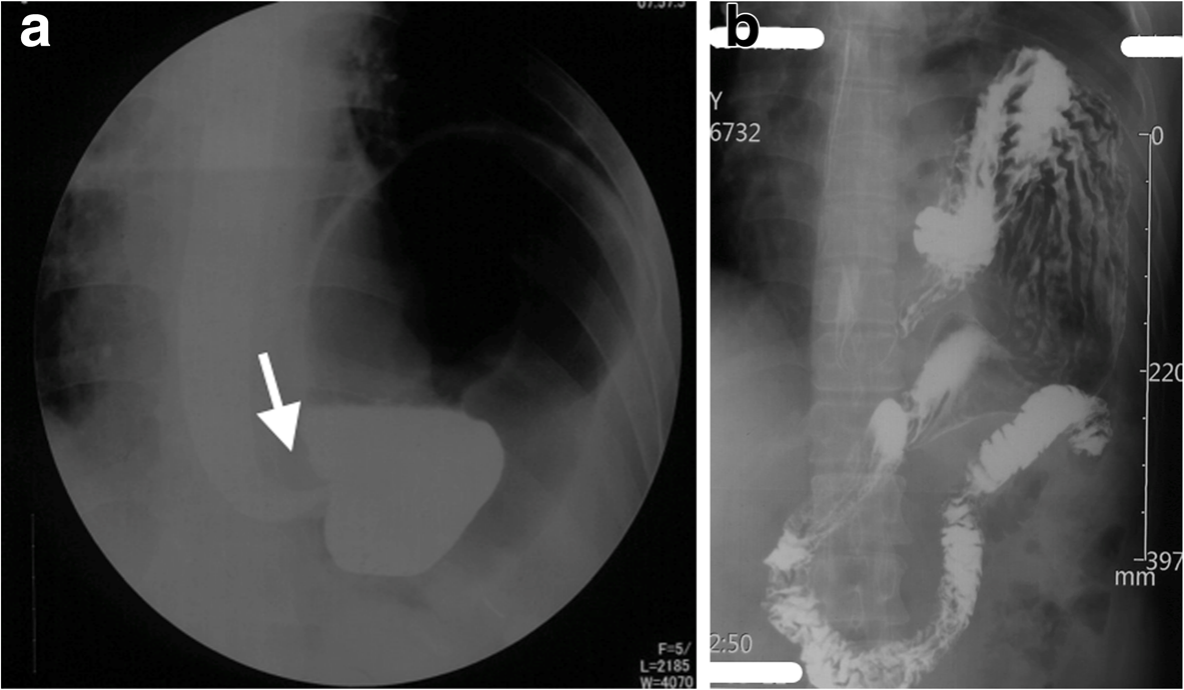
Upper digestive tract radiograph. **a** showed gastric volvulus with vertical gastroesophageal angle (the arrow). **b** showed the full view of the gastric volvulus

**Fig. 2:**
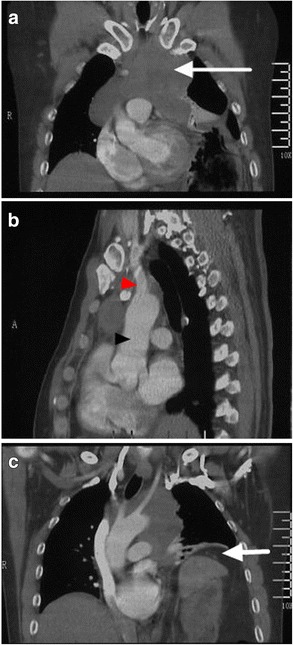
CT scan: Coronal and Sagittal reconstruction (**a**, **b**) showed an irregular mass in the anterior mediastinum, about 6.8 × 9.5 cm (the long arrows), surrounding the ascending aorta (the black arrowhead), and brachiocephalic trunk (the red arrowhead). Coronal reconstruction (**c**) showed elevated left diaphragm, twisted stomach, spleen, parts of bowels and gastric distension (the short arrow)

**Fig. 3:**
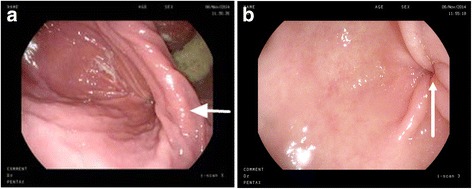
**a** Endoscopy view before de-rotation: longitudinal mucosa pitting, fundus dilatation and gastric body volvulus (the short arrow). **b** Endoscopy view after de-rotation. The pylorus showed up (the long arrow)

A 43-year-old man was admitted to our hospital for acute postprandial abdominal pain, nausea and vomiting, without dyspnea and myasthenia. There was no history of trauma. Physical examination found that trachea shifted to the right, breathing sound over the left lower chest decreased, cardiac border shifted to the right and tenderness in the upper abdomen. A chest X-ray showed that mediastinum and trachea shifted to the right and the left diaphragm lifted. Upper digestive tract radiograph (Fig. 1) showed left diaphragm elevated, gastric fundus and body lifted with nearly vertical gastroesophageal angle. A diagnosis of organoaxial gastric volvulus was suspected. On CT scan (Fig. 2), an irregular mass was found in the anterior mediastinum, about 6.8 × 9.5 cm, left diaphragm was elevated, stomach, spleen, parts of bowels were lifted. Emergency gastroscopy (Fig. 3) demonstrated gastric body volvulus. He was treated with endoscopic de-rotation.

The ultrasound guided biopsy of mediastinal mass was performed for this patient. The pathological findings revealed type B3 thymoma. According to the imaging examination he was diagnosed as Masaoka stage III thymoma. The thymoma of this patient presented with extensive pericardium, aorta arch and its branch infiltration, so it was considered as unresectable disease.

The patient with technically unresectable disease received radiotherapy from December 2013. Gastric volvulus still attacked about once a month, with the incentives of cold, satiety or spicy food. Each time endoscopic de-rotation was needed to relieve the symptoms. In 10 months later, CT scan showed the mass in the anterior mediastinum shrank, but a new small lesion developed on the left pleura. CT-guided biopsy of the pleural mass was performed. The histopathology and immunohistochemistry proved thymoma metastasis. The chemotherapy of carboplatin combined with paclitaxel was given for four cycles. He also received radiotherapy on the pleural mass. Disease was stable for 7 months. Gastric volvulus still occurred with less frequency of about every three to four months, and the symptoms were less severe. The left pleural effusion developed in May 2015. The disease was evaluated as progression. Then he received the chemotherapy of ADOC regimen (cisplatin, doxorubicin, vincristine, cyclophosphamide) for 2 cycles. Till March 2017, he was still alive with slow progression. The gastric volvulus still occurred every 3–4 months.

## Discussion and conclusion

Most patients with acute gastric volvulus present with a trio of symptoms known as Borchardt’s triad; these include pain in the upper abdomen or lower chest, retching and an inability to pass a nasogastric tube. Although the symptoms present in about 70% of patients with acute GV, diagnosis of gastric volvulus cannot be made by history and physical examination alone. Plain abdominal radiograph or computed tomography(CT) are recommended for accurate diagnosis. The classic finding of acute GV on plain radiograph is a single large spherical gas bubble located in the upper abdomen or chest with an air-fluid level [6]. Compared with plain radiography, CT has the added advantage of showing the relationship between the stomach and its surrounding structures helping identify any anatomic abnormalities associated with secondary GV [7]. All tests of chest radiograph, upper digestive tract radiograph and CT scan of our patient suggested GV, confirming the diagnosis of acute gastric volvulus.

For this patient, GV was considered secondary to thymoma. There was no history of GV or etiological factors such as previous trauma and GV only presented accompanied by thymoma. An underlying pathophysiologic mechanism related to phrenic nerve palsy may be responsible. The phrenic nerve (PN) has been shown to be involved in up to 33% of patients with advanced stage thymoma (Masaoka Stage III and IV) [5]. The B3 thymoma often shows invasive growth and extensive involvement of the PN [8]. Involved phrenic nerve results in PN palsy which can cause diaphragmatic dysfunction, left diaphragm elevation, gastrophrenic ligaments stretching, and stomach dislocation. All of these abnormalities followed similar physiological malformations that lead to GV. To the best of our knowledge, there has no report of thymoma initially presented with acute gastric volvulus.

GV is a life-threatening condition, thus prompt treatment is imperative after diagnosis. For patients with acute GV and good surgical risk, surgery is recommended [9, 10]. A less invasive approach can be used in patients who have medical comorbidities that preclude surgery. This approach consists of endoscopic de-rotation and gastric fixation. Our patient was diagnosed as Masaoka stage III thymoma which was considered unresectable and received radiotherapy as definitive treatment. He did not undergo surgical repair for GV because of incurable thymoma. He was treated with endoscopic de-rotation as the first choice. GV still attacked during treatment with less severity and reduced frequency. Endoscopic de-rotation was performed successfully every time when GV occurred. Roberto Caronna reported a 51-year-old woman with extensive peritoneal metastasis from advanced ovarian cancer [11]. She underwent cytoreductive surgery including subphrenic peritonectomy. A diaphragmatic hernia with an intrathoracic gastric volvulus developed four months after surgery. Emergency laparotomy was operated to repositioned the stomach in the abdominal cavity and the diaphragmatic breach was directly sutured. When a patient was diagnosed as acute GV with unresectable malignant tumor, the benefit and risk of the surgery of GV must be balanced. Whether surgical repair is needed for GV patients with malignant tumor is worth further investigation.

We report the first case of thymoma initially presented with acute GV. The pathological mechanism may be related to the phrenic nerve palsy. This case indicates that thymoma can cause phrenic nerve palsy, presented with rare acute GV. In addition, for patient with GV, secondary etiology should be considered for early diagnosis and therapy.

## Abbreviations

CT: Computed tomography
GV: Gastric volvulus
PN: Phrenic nerve
